# Gabapentin Enacarbil Extended‐Release Versus Placebo: A Likely Responder Reanalysis of a Randomized Clinical Trial

**DOI:** 10.1111/acer.14414

**Published:** 2020-07-31

**Authors:** Eugene M. Laska, Carole E. Siegel, Ziqiang Lin, Michael Bogenschutz, Charles R. Marmar

**Affiliations:** ^1^ From the Department of Psychiatry Center for Alcohol Use Disorder and PTSD New York University Grossman School of Medicine One Park Avenue New York New York 10016 USA; ^2^ Department of Population Health Biostatistics Division New York University Grossman School of Medicine New York New York USA

**Keywords:** Alcohol Use Disorder, Gabapentin Enacarbil Extended‐Release, Likely Responder Analysis, Precision Medicine

## Abstract

**Background:**

We reanalyzed a multisite 26‐week randomized double‐blind placebo‐controlled clinical trial of 600 mg twice‐a‐day Gabapentin Enacarbil Extended‐Release (GE‐XR), a gabapentin prodrug, designed to evaluate safety and efficacy for treating alcohol use disorder. In the original analysis (*n* = 338), published in 2019, GE‐XR did not differ from placebo. Our aim is to advance precision medicine by identifying likely responders to GE‐XR from the trial data and to determine for likely responders if GE‐XR is causally superior to placebo.

**Methods:**

The primary outcome measure in the reanalysis is the reduction from baseline of the number of heavy drinking days (ΔHDD). Baseline features including measures of alcohol use, anxiety, depression, mood states, sleep, and impulsivity were used in a random forest (RF) model to predict ΔHDD to treatment with GE‐XR based on those assigned to GE‐XR. The resulting RF model was used to obtain predicted outcomes for those randomized to GE‐XR and counterfactually to those randomized to placebo. Likely responders to GE‐XR were defined as those predicted to have a reduction of 14 days or more. Tests of causal superiority of GE‐XR to placebo were obtained for likely responders and for the whole sample.

**Results:**

For likely responders, GE‐XR was causally superior to placebo (*p* < 0.0033), while for the whole sample, there was no difference. Likely responders exhibited improved outcomes for the related outcomes of percent HDD and drinks per week. Compared with unlikely responders, at baseline likely responders had higher HDDs; lower levels of anxiety, depression, and general mood disturbances; and higher levels of cognitive and motor impulsivity.

**Conclusions:**

There are substantial causal benefits of treatment with GE‐XR for a subset of patients predicted to be likely responders. The likely responder statistical paradigm is a promising approach for analyzing randomized clinical trials to advance personalized treatment.

## Graphical abstract

Individuals with AUD likely to respond to Gabapentin XR were identified based on predictive baseline clinical features. Their responses were causally superior to a matched group assigned to placebo. At baseline, likely responders had more heavy drinking days, lower levels of depression and anxiety and higher levels of cognitive and motor impulsivity. In the whole sample, there were no between treatment differences. The likely responder statistical paradigm as a valuable approach to analyzing RCTs and for advancing precision medicine.

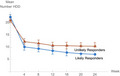

The search for medications to treat alcohol use disorder (AUD), a disorder reported to affect over 15 million persons in the United States, continues with high priority (https://pubs.niaaa.nihgov/publications/AlcoholFacts&Stats/AlcoholFacts&Stats.htm). Although there are 3 approved drugs for AUD, many individuals do not respond or even partially respond to any of them, motivating the search for new treatments (Litten, Falk, Ryan, Fertig, 2015). Unfortunately, it is not uncommon for randomized controlled clinical trials (RCTs) to find no difference between candidate therapies and placebo. However, quite commonly in RCTs some participants respond very well while others do not, leading to a small overall treatment effect. The hope for personalized medicine is that the particular features that make individuals more or less likely to respond can be identified and more specifically used to improve outcomes by matching treatments to patient characteristics. If this is possible for a test treatment, then the analysis of an RCT should identify likely responders (LRs) who are predictable from baseline features and perform causal inference on superiority to placebo in the subgroup.

Here, we report on the use of a LR analytic strategy in the reanalysis of a clinical trial of gabapentin enacarbil extended‐release (Falk and colleagues, 2019), a candidate treatment for AUD. In its immediate‐release form, gabapentin is approved by the FDA for the treatment of epileptic seizures, neuropathic pain, and restless leg syndrome (http://www.caremark.com/portal/asset/FEP_Rationale_Gabapentin.pdf). The clinical effects of gabapentin are thought to be mediated primarily by its high‐affinity binding to α_2_δ‐1‐containing voltage‐dependent calcium channels, although the exact mechanism is not clear (Sills and Rogawski, 2020). Gabapentin enacarbil extended‐release (GE‐XR) (HORIZANT^®^; Arbor Pharmaceuticals, LLC, Atlanta, GA) is a prodrug formulation of gabapentin. GE‐XR has shown promise for AUD based on both preclinical (e.g., Koob, 2008; Roberto et al., 2008) and clinical evidence (e.g., Myrick et al., 2009; Mason et al., 2014). Previous clinical trials have been inconsistent in the appraisal of the efficacy of gabapentin for treating AUD (Roberto et al., 2008; Anton et al., 2011; Mason et al., 2014; Falk et al., 2019). A recent meta‐analysis by Kranzler and colleagues (2019) found that there was evidence of a benefit only for 1 outcome variable, percentage of HDD (PHDD).

Falk and colleagues (2019) conducted a large multisite 26‐week randomized double‐blind placebo‐controlled clinical trial with a target dose of 600 mg of GE‐XR twice daily. Participants were 21 years of age or older, reported drinking an average of at least 21 standard drinks per week for women or 28 standard drinks per week for men, and had at least 1 heavy drinking day per week during the 28‐day period before consent and at least 3 consecutive days of abstinence prior to randomization. In the original analysis of the modified intent‐to‐treat sample, GE‐XR did not separate statistically from placebo on the primary endpoint, “zero heavy drinking days” evaluated during the final 4 weeks of the trial, nor on any other drinking measures. In discussing the results, Falk and colleagues (2019) cautioned that because of the “heterogeneity of the AUD population (Litten et al., 2015), average treatment effects do not sufficiently describe the efficacy of GE‐XR and that more nuanced moderator analyses are necessary to show efficacy among only certain participant subgroups.”

In this reanalysis of the RCT, we identified LRs to the treatment and utilized a causal potential outcome framework (Rubin, 1974, 2005; Imbens and Rubin, 2015) to test treatment efficacy. To identify LRs to GE‐XR, we used a machine learning method, random forests, to predict outcome from subject baseline features. The primary outcome measure is the change from baseline in the number of heavy drinking days during the maintenance phase, the last 4 weeks of the trial, denoted by ΔHDD. Likely responders are individuals whose *predicted* ΔHDD is 14 days or more.

## Materials and Methods

The detailed study profile and CONSORT flow diagram are presented in Falk and colleagues (2019).

### Participants

We used the modified intent‐to‐treat sample (*n* = 338) of Falk and colleagues (2019) comprised of persons who received at least 1 dose of the investigational drug of whom 170 were assigned to GE‐XR. Participants were treatment‐seeking volunteers with a DSM‐5 diagnosis of at least moderate AUD (*i*.*e*., 4 or more criteria) in the past year, were at least 21 years of age, reported drinking an average of at least 21 standard‐size drinks per week for women or 28 standard‐size drinks per week for men, had at least 1 heavy drinking day per week during the 28‐day period before consent and prior to randomization, and were abstinent for at least 3 consecutive days. As summarized in Table 1 of Falk and colleagues (2019), treatment groups did not differ statistically in baseline features including excessive drinking behaviors or symptoms of impulsivity, anxiety, depression, and mood disturbances.

**Table 1:** Baseline Characteristics. Unlikely Responders Compared to Likely Responders

Variable	Unlikely responders *n* = 197	Likely responders *n* = 141	Unadjusted *p*‐value^a^
Demographics			
Age (SD)	49.72 (10.99)	50.51 (10.65)	0.51
Gender (%)	Male (65.99)	Male (65.96)	1.0
Years of Education (SD)	15.37 (2.69)	15.07 (2.62)	0.308
Race (%)	White (76.65) Black (15.74)	White (65.25) Black: (22.70)	0.202
Drinking measures			
Percent HDD (%)	74.80 (23.29)	79.81 (21.21)	**0.044**
Drinks per week (SD)	54.71 (25.45)	59.21 (34.18)	0.166
Drinks/drinking day (SD)	9.06 (4.03)	9.68 (5.12)	0.215
Days abstinent (%)	12.65 (15.31)	12.21 (15.57)	0.794
Alcohol attitude measures			
Alcohol Craving Questionnaire (SD)	2.82 (0.79)	2.67 (0.89)	0.617
Alcohol‐related consequences (SD)	20.93 (9.30)	21.77 (11.45)	0.456
AUD symptoms (SD)	7.61 (2.06)	7.11 (2.15)	**0.033**
AUD severity (% Severe)	79.19	68.79	**0.041**
Motivation to reach goal (SD)	8.73 (1.49)	8.99 (1.42)	0.115
Confidence to reach goal (SD)	6.86 (2.33)	6.62 (2.33)	0.340
Behavioral measures			
BIS—Attention (SD)	15.58 (4.24)	15.07 (4.16)	0.270
BIS—Motor (SD)	21.97 (3.61)	23.07 (4.23)	**0.011**
BIS—Nonplanning (SD)	23.00 (5.48)	24.23 (5.78)	**0.048**
Beck Anxiety Inventory (SD)	8.25 (7.47)	5.86 (7.84)	**0.005**
Beck Depression Inventory (SD)	11.49 (8.64)	9.09 (8.57)	**0.012**
Profile of Mood States Scale (SD)	63.87 (20.18)	55.81 (15.73)	<**0.001**
CIWAA—R^b^ (SD)	1.69 (2.90)	1.20 (1.84)	**0.074**

BIS, Barrett Impulsiveness Scale.

^a^ *p*‐values based on chi‐square or Wilcoxon rank tests as appropriate. Values shown in bold indicate substantial evidence of a difference between the likely and unlikely responder groups.

^b^ Clinical Institute Withdrawal Assessment of Alcohol—R.

### Primary Outcome Measure

The primary outcome measure specified by Falk and colleagues (2019) was zero heavy drinking days during the last 4 weeks of the maintenance phase of the study (weeks 22 to 25). A heavy drinking day is defined as 4 or more standard drinks for women and 5 or more drinks for men. While reduction to zero is clearly desirable, it is a binary outcome measure, achieved by only about one‐quarter of participants receiving GE‐XR. We used ΔHDD as the primary outcome measure, which provides a broader characterization of change in drinking behavior with greater statistical power.

### Missing Data

There were 166 participants with missing items scattered across the clinical feature data during the last 4 weeks of the study, 141 of whom were missing only 1 item, 23 were missing 2, and 2 were missing fewer than 5. Fifty‐eight were missing the number of HDD in the last 4 weeks. Falk and colleagues (2019) imputed PHDD and percentage of days abstinent by assigning individual days with missing drinking data as heavy drinking days and drinking days, respectively. To impute the missing values on all variables, we used a method based on chained equations, using the software R package mice (Buuren and Groothuis‐Oudshoorn, 2010).

### Definition of Likely Responders

In this study, the number of HDD that a subject can have at baseline ranges from 0 to 28. A reduction in HDD of 14 days or more guarantees that the response is at least 50% of the baseline. This criterion is consistent with a commonly used definition of “response” of a 50% or greater reduction from baseline severity of a relevant measure. For this reason, individuals are called LRs to GE‐XR if, based on baseline features, their *predicted* ΔHDD ≥ 14. Note that individuals identified as LRs are predicted to have an outcome that meets the criterion but, in fact, based on their actual change in the trial, they may or may not meet this criterion. Individuals who are not LRs are called unlikely responders (URs) and similarly, their actual ΔHDD may be ≥ 14.

### Prediction Function of Treatment Response, ΔHDD to GE‐XR, Used to Define Likely Responders

To predict the change in the number of drinking days to GE‐XR for an individual with baseline features *f*, denoted Pred[ΔHDD| GE‐XR, *f*], we used a random forest (RF) model in regression tree mode (Breiman, 2001). The model, based on prerandomization features of the participants, was fit on the individuals assigned to GE‐XR and subsequently applied to all subjects regardless of treatment assignment or actual outcome to obtain an estimate of their expected response to GE‐XR. Baseline predictive features used in the model include demographics, substance use indicators, and psychiatric characteristics. Individual items rather than summary scores were used for each scale. RF uses bootstrap sampling, and estimates of the properties of the model fit are obtained from those not selected, the designated “out‐of‐bag” sample. The goodness of fit of the model was measured by the square of the correlation between the observed and model‐predicted values, which may be considered an estimate of the percentage of the variance accounted for by the RF model.

### Testing Causal Superiority of GE‐XR to Placebo for Likely Responders and for the Whole Sample

We employ a potential outcome framework (Neyman, 1923; Rubin 19^74^, ^2005^; Imbens and Rubin, 2015) which starts with the premise that a causal effect is based on a comparison of an individual’s potential outcomes from receiving a treatment and a control. These are the theoretical responses that would have resulted had the individual been assigned to one of the treatments, the clock rolled back, and the individual assigned to the other treatment. An individual’s causal treatment effect is defined as the difference between the two potential outcomes. In an RCT, subjects are randomized to only one of the two treatments: one outcome is observed, and the other is said to be counterfactual. The average causal treatment effect (ATE) in a group is the average of the individual potential outcomes of members of the group. Under the potential outcome framework, randomization enables valid estimation of the ATE in the whole sample allowing causal statements to be made. Another method essential for causal inference particularly for small samples is to match subjects on baseline measures that are predictors of outcome. Hansen (2008) has shown that instead of the tedious task of matching on baseline covariates, it suffices to match on a balancing score to achieve equal distributions of predictor variables. Similar to a propensity balancing score in observational studies (Rosenbaum and Rubin, 1983), Pred[ΔHDD| GE‐XR, *f*] is a prognostic balancing score (Hansen, 2008) which in the current setting is estimated by the RF for every subject. These scores were rank‐ordered and divided into quantiles, the number of which depends on the total sample size of the group. Two quantiles were used for the LR subjects and five were used for the whole sample. In every quantile, there are some subjects who were assigned to GE‐XR and some to placebo and the two groups have approximately the same distribution of prognostic variables.

To test for treatment differences within quantiles, a linear regression model was fit for the LRs and separately for the whole sample with terms for treatment assignment, baseline HDD, quantile, and treatment‐by‐quantile interactions. Goodness‐of‐fit statistics for the models were obtained.

Closed testing was used to control the family‐wise error rate. There are two primary hypotheses in this reanalysis. The first primary null hypothesis is there are no treatment differences in the LR group and the second is there are no differences in the whole sample. The first hypothesis was tested at a type 1 error bound of 0.05. If the hypothesis is not rejected, testing is ended. If the null for LRs is rejected, the null hypothesis for the total study sample is tested. All other hypothesis tests in all of the tables are displayed at their nominal *p*‐value for use in interpretation of the magnitude of the observed effects relative to the standard deviation. No correction was made for multiplicity except in the testing of the two primary hypotheses.

Analyses of other AUD and behavioral outcome measures over the last four weeks of the study were conducted to examine whether there are differences in outcome between treatments (i) among LRs and (ii) among URs and whether there is a difference in the LR group compared to the UR group (iii) for GE‐XR outcomes and iv) for placebo outcomes. For each outcome, a 1‐way ANOVA with terms for treatment and responder group was used. Pairwise contrasts were performed comparing GE‐XR and placebo within the responder groups, comparing the response to GE‐XR between the 2 responder groups, and comparing the response to placebo between the 2 responder groups.

### Estimating The Effect of Risk Factors

To understand the extent to which a specific baseline feature, x, affects the random forest prediction of Pred[ΔHDD|GE‐XR, f*, x], where *f* * represents all other features in the model, we evaluate the average change in prediction caused by an increment of 1 unit of change, δ, in feature x, leaving all other features unchanged. Estimates of Pred[ΔHDD|GE‐XR, *f* *, x] and Pred[ΔHDD|GE‐XR, *f* *, x + δ] are obtained from the random forest for every subject in the trial. The median difference across individuals is an estimate of the change in expected outcome of an increment of δ in feature x for individuals treated with GE‐XR. The median was used instead of the mean because of the skewness in the distribution of the predictions. Consistent with epidemiological contexts, this can be called a risk difference. Risk differences were obtained for the features identified as predictors in the RF algorithm. To study whether incremental risks were additive or synergistic, changes induced by 2 items simultaneously were also examined.

## Results

In the total sample, there were 141 individuals predicted on the basis of their baseline characteristics by the RF to be LRs to GE‐XR, of whom 67 had been randomized to GE‐XR. LRs were grouped into 2 quantiles of 70 and 71 subjects per quantile. The whole sample of participants in the RCT were grouped into 5 quantiles based on their predicted response to GE‐XR with 67 or 68 subjects in each quintile.

### Baseline Characteristics

**Fig. 1:**
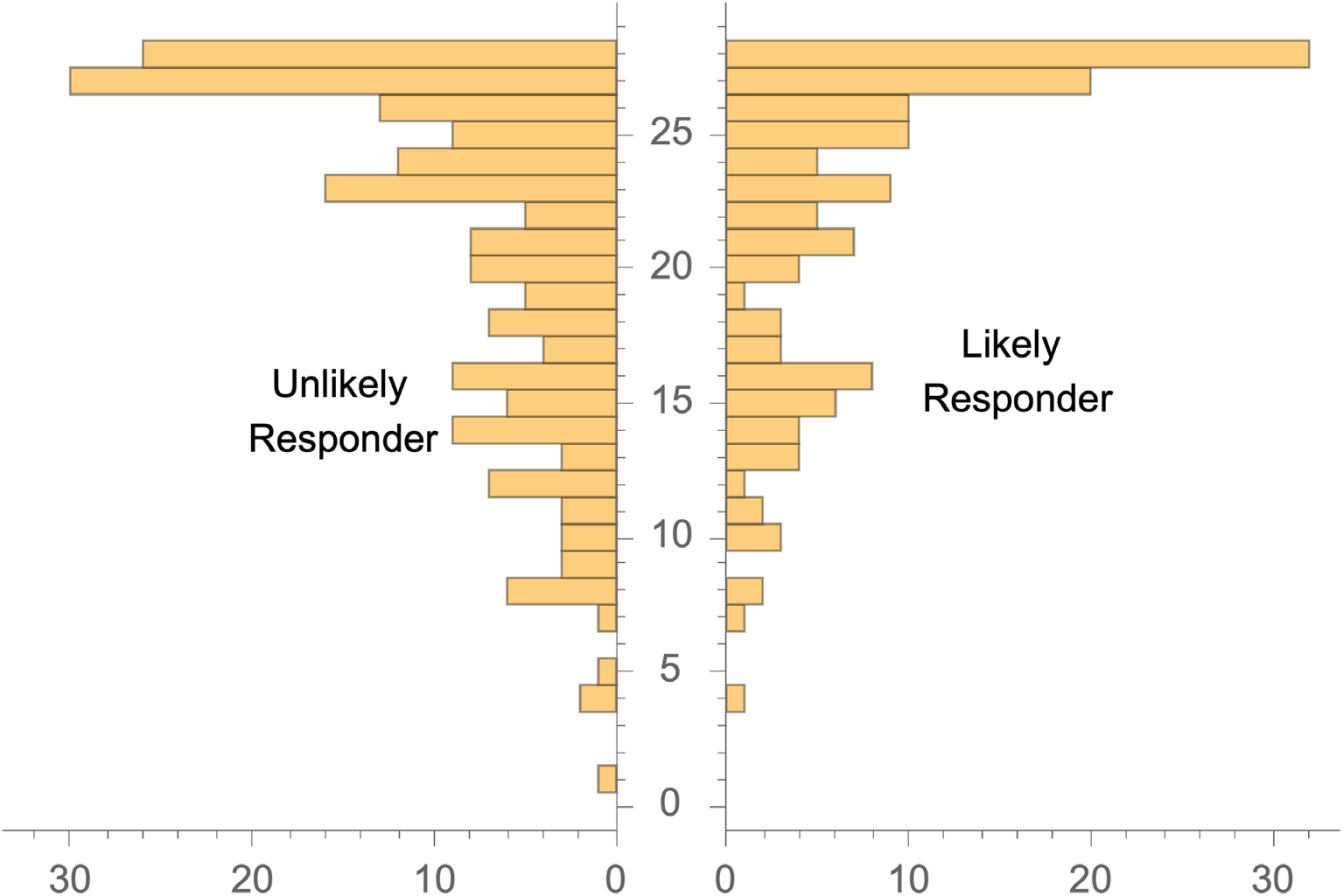
Histogram of baseline HDD for likely and unlikely responders.

Table 1 displays the mean value of baseline characteristics of LRs compared to URs. Figure 1 displays a paired histogram of the number of HDD at baseline for LRs and URs. The LR group had a slightly higher mean number of HDD (22.35, SD 5.94) than the UR group (20.94, SD 6.52), *p*‐value 0.044. LRs had somewhat milder symptoms than the URs on alcohol as well as on all of the anxiety, depression, and mood scales. On the other hand, in comparison with URs, LRs had higher impulsivity scores for motor and nonplanning activities.

### Model Fit of Random Forest Prediction of ΔHDD

**Fig. 2:**
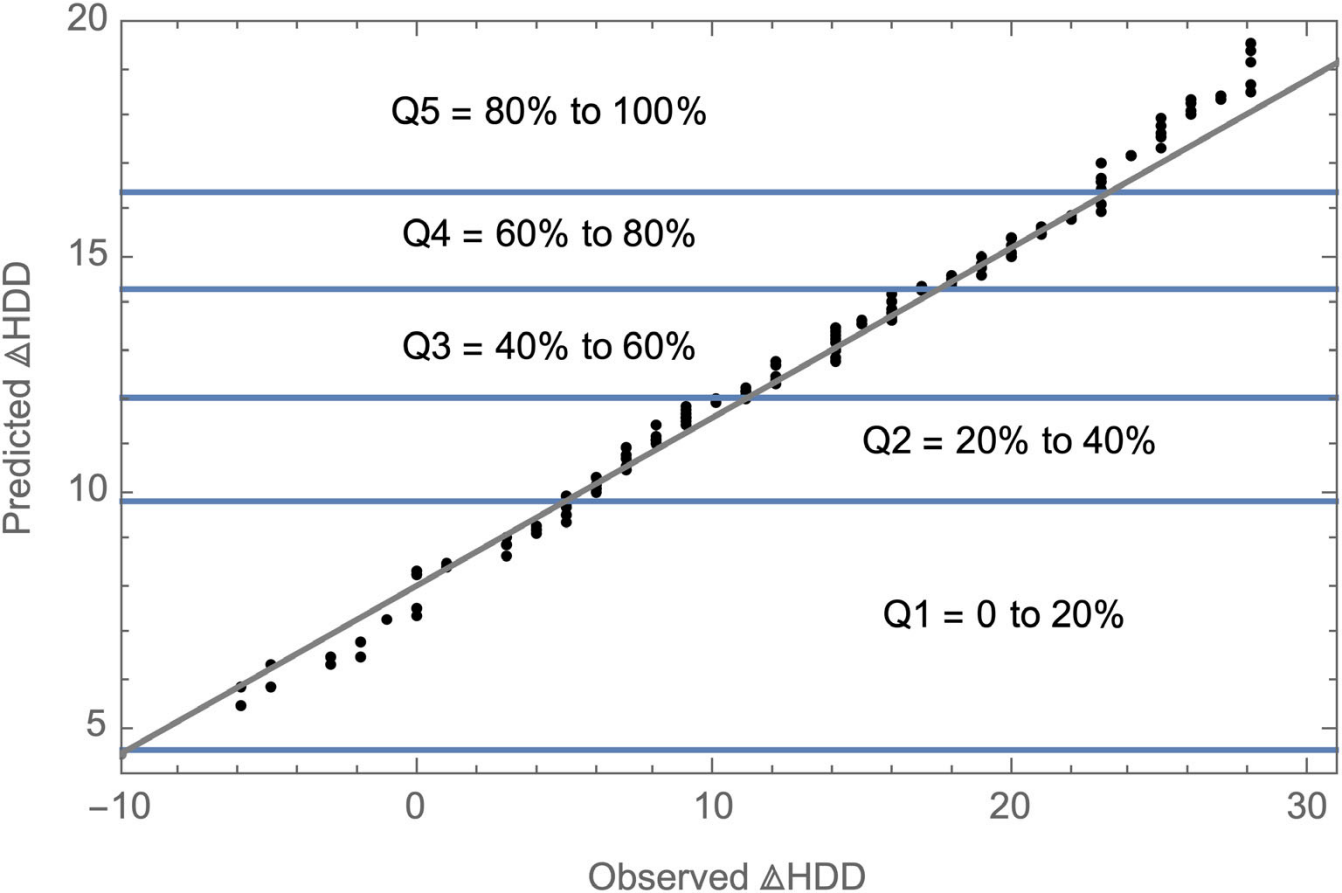
Q‐Q plot. Observed vs predicted ΔHDD for the GE‐XR sample.

A random forest in regression mode was used to estimate ΔHDD given treatment GE‐XR and baseline features *f*. Figure 2 displays the Q‐Q plot of the observed versus the RF‐estimated values of ΔHDD for the GE‐XR participants. The fit in quantiles 2, 3, and 4 is good, as evidenced by the proximity of plotted points to the 45‐degree line. In quintile 1, the treatment outcome is slightly overestimated, and in quintile 5, it is slightly underestimated. The Pearson correlation between observed and estimated values of ΔHDD is 0.415 with a 95% confidence interval of (0.30, 0.52). By analogy with regression theory, the percent of the variance explained by the model (the square of the correlation coefficient) is 17.22%.

### Treatment Effectiveness

#### Mean Number of HDD Over the Course of the Trial

**Fig. 3:**
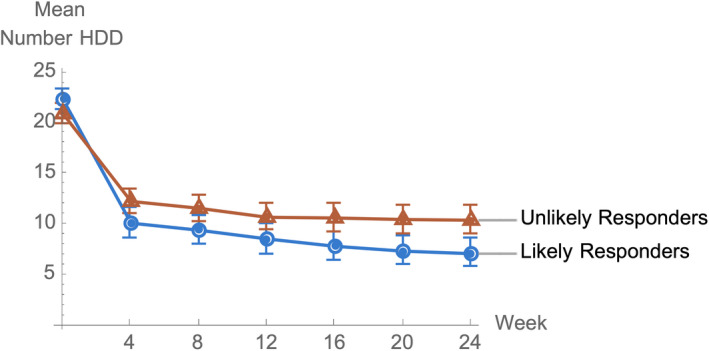
Mean number of monthly HDD for those randomized to GE‐XR over time by likely responder status.

The observed monthly mean number of HDDs for LRs and for the remaining URs for individuals randomized to GE‐XR, together with 95% confidence limits, is shown in Fig. 3. The means of the 2 responder groups begin to separate in the first month after treatment, with nonoverlapping confidence intervals in the last 2 months. The mean number of HDDs for LRs decreases slightly from month to month, whereas the slope for the URs flattens by the third month.

#### Likely Responder Analysis of Treatment Differences for ΔHDD

For the LR regression model, the correlation between observed and predicted treatment differences is 0.53 (CI, 0.42, 0.62) and the percent of the variance explained is 28.09%. The results of the analysis of treatment differences are presented in Table 2, which lists the mean predicted and observed data as well as the results of the test of treatment differences. LRs had an average model‐predicted ΔHDD due to GE‐XR of over 16 days and those randomized to GE‐XR had an observed value of almost 18 days. The regression model treatment difference was 4.12 days with a 95% CI (0.42, 7.83) and pooled *p*‐value of 0.031. The placebo group response was lower than the GE‐XR response in both quantiles as well.

**Table 2:** Average Treatment Effects of Likely Responders Within Quantiles Defined by Predicted ΔHDD Response to GE‐XR

Quantiles of Predicted ∆HDD	Treatment	*N*	Range of predicted ∆HDD for GE‐XR^a^	Mean observed ∆HDD^b^	Mean observed treatment difference^c^ (SD)	Model‐estimated treatment difference (95% CI)	Unadjusted *p*‐value test of treatment effect^d^
1	GE‐XR	34	14.00‐16.12	16.62	4.03 (2.14)	4.31 (0.53, 8.09)	**0.029**
	Placebo	36		12.58			
2	GE‐XR	33	16.16‐21.03	18.79	5.31 (1.93)	4.41 (0.86, 7.96)	**0.018**
	Placebo	38		13.47			
Pooled 1‐2	GE‐XR	67	14.00‐21.03	17.71	4.68 (1.44)	4.12 (0.42, 7.83)	**0.031**
	Placebo	74		13.03			

^a^ The within‐quantile range of the predicted response, ΔHDD, to treatment with GE‐XR based on the random forest prediction model. Estimates included in the range for the patients assigned to placebo are counterfactual because they never received GE‐XR.

^b^ Mean of the observed ΔHDD for each of the 2 treatments.

^c^ Mean of the observed difference between treatments in ΔHDD.

^d^ Bolded *p*‐value values indicate substantial evidence of a difference between the treatments. The within‐quantile unadjusted *p*‐values are intended to give an indication of the quantile effect size and not the results of hypothesis testing. The primary analysis, a test of treatment differences for the primary outcome measure is displayed in the last row based on pooling across the 2 quantiles.

#### *Full Sample Analysis of Treatment Differences for* Δ*HDD*

For the regression model of treatment response for the whole sample, the correlation between predicted and observed difference is 0.52 (CI, 0.46, 0.59) and the percent of the variance explained is 27.04%. The results of the treatment difference analysis for the whole sample are presented in Table 3. The observed mean response rate for GE‐XR for the whole sample is 13.16 compared to 11.96 for placebo. Based on the pooled quintile statistics, the treatment effect size (treatment difference divided by the standard deviation) is only 0.89, which does not offer statistical evidence of a difference in treatments when averaged over the whole sample. A model fit for analyzing the data without a quintile decomposition resulted in a similar overall effect of 0.71, 95% CI (−1.15, 2.58), also failing to provide statistical evidence of a treatment difference. For LRs who fell in quintiles 4 and 5 in which GE‐XR had a greater observed mean ΔHDD than placebo, the resulting *p‐*values were 0.011 and 0.033. Placebo was superior to GE‐XR in quintile 1, but there were only small treatment differences in quintiles 2 and 3.

**Table 3:** Average Treatment Effects of the Full Sample Within Quantiles Defined by Predicted ΔHDD Response to GE‐XR

Quintiles of Predicted ∆HDD)	Treatment	N	Range of predicted ∆HDD for GE‐XR^a^	Mean observed ∆HDD^b^	Mean observed treatment difference^c^ (SD)	Model‐estimated treatment difference (95% CI)	Unadjusted *p*‐value test of treatment effect^d^
1	GE‐XR	35	4.54 to 9.81	7.371	−4.48 (2.00)	−4.62 (−8.51, −0.73)	**0.023**
	Placebo	33		11.848			
2	GE‐XR	35	9.83 to 12.03	10.343	−1.47 (2.30)	−1.99 (−6.14, 2.16)	0.352
	Placebo	32		11.812			
3	GE‐XR	36	12.03 to 14.32	12.667	1.92 (2.53)	1.46 (−3.13, 6.04)	0.536
	Placebo	32		10.75			
4	GE‐XR	32	14.34 to 16.37	16.469	4.73 (2.16)	5.11 (1.27, 8.94)	**0.011**
	Placebo	35		11.743			
5	GE‐XR	32	16.41 to 21.36	18.938	5.27 (1.99)	4.07 (0.40, 7.73)	**0.033**
	Placebo	36		13.667			
Pooled 1‐5	GE‐XR	170	4.54 to 21.36	13.16	1.94 (0.99)	0.89 (−3.70, 5.48)	0.704
	Placebo	168		11.96			

^a^ The within‐quantile range of the predicted response, ΔHDD, to treatment with GE‐XR based on the random forest prediction model. Estimates included in the range for the patients assigned to placebo are counterfactual because they never received GE‐XR.

^b^ Mean of the observed ΔHDD for each of the 2 treatments.

^c^ Mean of the observed difference between treatments in ΔHDD.

^d^ Bolded *p*‐value values indicate substantial evidence of a difference between the treatments. The within‐quintile unadjusted *p*‐values are intended to give an indication of the quintile effect size and not the results of hypothesis testing. The full sample test of treatment differences is displayed in the last row based on pooling across the 5 quintiles.

#### Analysis of Other AUD and Behavioral Outcome Measures

Table 4 provides a comparison of treatment differences within and between the 2 responder classes for other AUD and behavioral measures. For within‐responder group comparisons on other AUD measures, only drinks per week among LRs showed any evidence of an advantage of GE‐XR (15.67, SD 15.91) over placebo (22.57, SD 23.26) with an uncorrected *p*‐value of 0.061. On the same measure in the UR group, placebo (21.66, SD 17.31) is superior to GE‐XR (29.54, SD 22.94) with a *p*‐value of 0.015. There are no other between‐treatment differences in either the LR or the UR group for any other measures. As for comparisons of the same treatment across the 2 responder groups, placebo mean response is about the same for all measures in the table. However, in comparing LR to UR outcomes for GE‐XR, the average outcome in the UR group for AUD measures is worse than in the LR group with small *p*‐values for 3 of the 5 measures. Similarly, for both the Beck Depression and Anxiety Inventories there is substantial difference in outcomes for the UR group versus the LR group among those randomized to GE‐XR, with those in the UR showing more severe symptomatology.

**Table 4:** AUD and Behavioral Outcome Measures at Study End: Comparisons of Treatment Response Within and Between Responder Groups^a^

Variables	Comparisons within unlikely responders (URs)			Comparisons within likely responders (LRs)			Comparisons between responder groups	
	G^b^	P^b^	*p*‐value^c^ G vs P UR	G	P	*p*‐value^d^ G vs P LR	*p*‐value^e^ G UR vs LR	*p*‐value^f^ P UR vs LR
Percent HDD	40.08 (34.07)	34.00 (32.49)	0.203	17.75 (24.06)	32.24 (33.18)	**0.004**	**<0.001**	0.73
Drinks per week	29.54 (22.94)	21.66 (17.31)	**0.015**	15.67 (15.91)	22.57 (23.26)	**0.061**	**<0.001**	0.794
Drinks per drinking day	4.44 (4.01)	3.87 (3.22)	0.272	3.63 (2.82)	3.53 (3.86)	0.863	0.15	0.534
Percent of days abstinent	32.81 (33.62)	41.18 (34.41)	0.118	52.02 (32.99)	42.37 (36.99)	0.139	**<0.001**	0.849
Percent of subjects abstinent	10.81 (9.64)	11.43 (10.12)	1.0	10.34 (9.27)	13.73 (11.84)	0.805	1.0	0.921
Alcohol Craving Questionnaire	2.59 (0.85)	2.51 (0.81)	0.544	2.53 (0.74)	2.68 (1.11)	0.416	0.687	0.322
Alcohol‐related consequences	10.31 (9.63)	9.81 (8.70)	0.746	7.98 (8.61)	9.04 (9.51)	0.544	0.151	0.643
Beck Depression Inventory	7.35 (7.80)	6.11 (7.11)	0.322	3.79 (5.51)	4.27 (6.94)	0.688	**0.004**	0.158
Beck Anxiety Inventory	5.88 (7.48)	4.16 (5.63)	0.123	2.45 (4.93)	3.00 (5.65)	0.587	**0.003**	0.267
Profile of Mood States Scale	59.76 (22.72)	59.19 (18.37)	0.869	54.07 (15.58)	54.84 (22.73)	0.834	0.106	0.248

^a^ These analyses are based on a 1‐way ANOVA with terms for treatment and responder groups. Hypothesis tests are based on pairwise contrasts.

^b^ G = GE‐XR, P = placebo. Shown are mean values and SD.

^c^ The *p*‐values for the within‐responder group pairwise contrasts between GE‐XR and placebo for the UR group.

^d^ The *p*‐values for the within‐responder group pairwise contrasts between GE‐XR and placebo for the LR group.

^e^ The *p*‐values for the between‐responder group pairwise contrasts for GE‐XR.

^f^ The *p*‐values for the between‐responder group pairwise contrasts for placebo.

### Impact of Risk Factors on Predicted Change in HDD

Table 5 displays the results of the evaluation of the average predicted ΔHDD for individuals treated with GE‐XR caused by an increment of 1 unit of change in a specific feature leaving all other features unchanged. The 2 largest changes occur with BIS question 12, “I am a careful thinker,” improving predicted outcome by 2.2 days, and IMB question 14, “My family/friends have been hurt,” improving predicted outcome by 1.37 days. An increase of 1 unit in the BIS question implies a decrease in thoughtfulness and in the IMB question implies an increased frequency of hurting friends. These attributes predict a better outcome. All other predictors cause minimal decrease in ΔHDD of less than 1 day. Examining predictors in pairs suggested an additive effect.

**Table 5:** Change in Predicted ΔHDD for GE‐XR Patients Resulting From a Unit Increase in Important Baseline Predictor Variables^a^

Scale: item number	Item	Numerical value of item categories	Mean change in predicted ΔHDD for a unit increase in item^b^
BIS: Q12	I am a careful thinker	1‐4: almost always to never; 5: refuse to answer	2.20 (*p*‐value < 0.001) (2.07, 2.34)
IMB: Q14	My family/friends have been hurt	0‐4: never to all the time	1.37 (*p*‐value < 0.001) (1.21, 1.54)
BAI: Q4	Bothered by unable to relax	0‐3: not at all to severe	‐0.85 (*p*‐value < 0.001) (−0.96, −0.73)
POMS: Q18	Blue	0‐4: not at all to extremely	‐1.23 (*p*‐value < 0.001) (−1.36, −1.09)
AUD: Q10	Did you continue to use alcohol?	No = 0 Yes = 1	‐0.56 (*p*‐value 0.058) (−0.83, −0.32)
POMS: Q26	Uneasy	0‐4: not at all to extremely	‐0.90 (*p*‐value < 0.001) (−1.05, −0.76)
POMS: Q27	Restless	0‐4: not at all to extremely	‐0.41 (*p*‐value < 0.001) (−0.55, −0.27)
BIS: Q9	I concentrate easily	1‐4: almost always to rarely	‐0.46 (*p*‐value < 0.018) (−0.60, −0.34)

ACQ, Alcohol Craving Questionnaire; AUD, alcohol use disorder; BAI, Beck Anxiety Inventory; BIS, Barrett Impulsiveness Scale; IMB, I imbibe; POMS, Profile of Mood States; PSQI, Pittsburgh Sleep Quality Index.

^a^ Shown are predictor variables for which a unit increment resulted in a change in predicted ΔHDD of at least ± 0.4.

^b^ Shown in a row are the result of a unit increase in the item on ΔHDD, the unadjusted *p*‐values resulting from a chi‐square test comparing the UR and LR groups at baseline, and a 95% confidence interval for the change obtained from the random forest.

## Discussion

In a meta‐analysis of gabapentin for AUD, Kranzler and colleagues (2019) reported limited benefits and called for additional studies to define more clearly the role of gabapentin in AUD treatment. We reanalyzed a multisite 26‐week randomized double‐blind placebo‐controlled clinical trial (Falk et al., 2019) of 600 mg twice‐a‐day gabapentin enacarbil extended‐release, a gabapentin prodrug, designed to evaluate safety and efficacy in reducing heavy drinking in alcohol use disorder. In the original analysis, GE‐XR did not separate from placebo. In our reanalysis of this trial, we utilized pretreatment clinical features of the participants and a random forest model in regression tree mode (Breiman, 2001) to identify a subgroup of LRs to GE‐XR. We demonstrated that GE‐XR is causally superior to placebo in the LR group.

To advance precision medicine, it is necessary to determine which patients are likely to benefit from a given medication, and which are not. We found that LRs to GE‐XR, compared with URs, prior to randomization had a higher number of HDDs, lower levels of anxiety, depression, and general mood disturbances, and higher levels of cognitive and motor impulsivity. These findings suggest that AUD patients with lower levels of internalizing symptoms such as anxiety and depression, and higher levels of externalizing problems including greater cognitive and motor impulsivity respond better to the gabapentin prodrug. Gabapentin is an anticonvulsant used to treat partial seizures, neuropathic pain, and fibromyalgia and restless leg syndrome. It is a gabapentinoid originally designed to be an analogue of the inhibitory neurotransmitter GABA. It does not bind to GABA receptors, but rather acts as a ligand at the α2δ‐1 subunit site of certain voltage‐dependent calcium channels. It is used off‐label as a mood stabilizer to modulate arousal, anger, and impulsivity in psychiatric disorders including bipolar disorder, PTSD, and borderline personality disorder as well as alcohol and drug withdrawal and craving. Its anticonvulsant‐related mood‐stabilizing properties may explain why GE‐XR is selectively effective for AUD patients with greater cognitive and motor impulsivity.

It was not a surprise that based on the whole RCT sample, there was no evidence of a treatment effect as reported in Falk and colleagues (2019). Nor is it surprising that there is a reversal of treatment effects in the UR group, where placebo has a better outcome than GE‐XR. Trials that report no overall difference in outcome may be due to divergent treatment effects among subgroups that cancel each other out. In Table 4, the individuals in the first quintile have better outcomes on placebo, those in quintiles 4 and 5 have better outcomes on GE‐XR, and those in quintiles 2 and 3 are agnostic. When pooled, there is scant evidence that the population average treatment effect differs from zero.

There is a long and extensive history of efforts to identify predictors of response and/or subgroups likely to respond to AUD treatments. Project MATCH, a very large NIAAA‐sponsored trial contrasting effects of cognitive behavioral therapy, motivational enhancement therapy, and 12‐step facilitation therapy, was designed to test for matching effects, but found few significant matching variables, although low psychiatric severity did predict better outcomes with 12‐step facilitation therapy (Project Match, 1997, 1998). Alcoholism typologies appear to have some influence on response to selective serotonin uptake inhibitors on drinking outcomes (Pettinati et al., 2000; Kranzler et al., 1996; Chick et al., 2004). Single genetic polymorphisms have been shown to influence response to ondansetron (Johnson et al., 2011) and topiramate (Kranzler, Feinn, Morris, Hartwell, 2019), while studies of the effect of the Asn40Asp polymorphism of the mu opioid receptor gene have been nonconclusive (Hartwell et al., 2020). A secondary analysis by Hou and colleagues (2015) of the Johnson and colleagues (2011) study referenced above used reduction of baseline PHDD (ΔPHDD, i.e., change in average PHDD during treatment period relative to baseline) as the primary outcome. Predictors included genetic polymorphisms and baseline clinical characteristics. They used machine learning methods to identify subgroups with reductions in heavy drinking days that were larger with ondansetron treatment than with placebo. A method called virtual twins (Foster et al., 2011) uses random forests with treatment as a covariate to estimate the pair Pred[ΔPHDD|GE‐XR, *f*] and Pred[ΔPHDD| placebo, *f*] for every subject. The statistical analysis treats the difference in these 2 predictors as outcome of the trial. None of the methods used to analyze these studies were designed to produce causal statements about treatment effects in subgroups of individuals who are identified as LRs.

The European Medicines Agency advises that “In every submission process of a new drug, subgroup analyses are mandatory in order to “check that the estimated overall effect is broadly applicable to relevant subgroups” (European Medicines Agency, 2019). Our view is that a precision medicine likely responder approach to the analysis of a RCT as used in this study may be more appropriate. That is, the primary analysis should first identify or, perhaps based on previous results, confirm the identity of individuals who are LRs based on baseline measures including biomarkers. These are the group of patients in the target population for whom the drug is likely to produce positive results and the group for whom treatment with the medicine is likely to be justified. The second step is to check causal superiority to the comparator in the LR group. Only in exceptional cases would a treatment be appropriate for an individual not likely to be a responder.

For the causal claim of differences between treatments to be valid, the definition of responder based on predicted response must be made in advance. Clearly, this is not the case in our reanalysis. The Falk and colleagues (2019) study, together with parameter estimates of the distribution of outcome measures and the binary “zero heavy drinking days” declared as the primary outcome, was published before we began the reanalysis. Although the FDA recommends responder‐based endpoints, including abstinence or no heavy drinking days, our primary outcome measure is ΔHDD and membership in the LR subset is achieved if the predicted ΔHDD ≥ 14 days. This is consistent with the finding of a recent review of ongoing clinical trials for AUD medications registered on ClinicalTrials.gov (Wallach et al., 2020) that there may “be a growing preference for end points that focus on reduced alcohol intake, which can lead to improved health‐related outcomes.” Despite some subjects having baseline HDD below 14, our choice of criterion was in consideration of the commonly used definition of a responder as a change from baseline of at least 50%. If predicted ΔHDD ≧ 14, the subject must have a percent reduction ≧ 50%. The entry inclusion criteria required only 1 HDD per week (4 per 28 days), but the mean percent HDD at baseline was 21.7 days. There were only 14 (9.5%) individuals with less than 14 days at baseline who received GE‐XR and 18 (13.5%) who received placebo. These subjects could have been dropped from our reanalysis, but this would violate the intent‐to‐treat principle. We examined the mean baseline HDD for each treatment in the LR group (GE‐XR: 16.26, SD: 1.65; placebo: 16.34, SD: 1.41) and in the UR group (GE‐XR: 11.41, SD: 1.85; placebo: 10.96, SD: 2.07) and found no within‐responder group treatment difference.

We could have used predicted percent reduction ≧50% as the definition of a LR. But it produces a different outcome as a function of baseline HDD for the same predicted ΔHDD. For example, those with a predicted reduction at study end of 13 ΔHDD who have 28 HDD at baseline score < 50% are therefore considered nonresponders, while those with the same predicted 13 ΔHDD who had 26 HDD at baseline score> 50% are therefore considered responders. Our definition is not without its own shortcoming. It would classify an individual with predicted ΔHDD of 13 as a nonresponder even if they achieve total abstinence.

As with any analysis of clinical trial data, the interpretation of these causal claims is also limited by the characteristics of the sample. The Falk and colleagues (2019) study required 3 days of abstinence at randomization, so those who were highly physically dependent were excluded. Also, less than 10% of the sample had a treatment goal of abstinence from alcohol. Findings might not generalize to patients with an abstinence goal. Those with current psychiatric illness and other substance use disorders were also excluded, complicating the interpretation of the finding that LRs to GE‐XR had lower levels of anxiety and depression.

It is important to caution that the potential outcomes enriched subsample approach require a well‐calibrated predictive model. In this analysis, the model fit was reasonably good, but there may be better models and other predictive features that would enable increased predictive accuracy. The RF model we found may not generalize to a broader population. Replication remains the cornerstone of increasingly convincing evidence.

## Conclusions

Focusing on the enriched subsample of LRs and applying potential outcomes methods in the analysis of a RCT are a potentially important advance for finding treatments for the population with AUD. The reanalysis shows that in a trial that failed to show overall superiority of GE‐XR to placebo across several alcohol drinking outcomes, a subset of the sample can be identified by their baseline clinical features who are LRs to the active treatment. They are individuals whose predicted reduction in HDD was greater than 2 weeks. Compared to URs, at baseline LRs had greater heavy drinking days, but lower levels of depression and anxiety and higher levels of cognitive and motor impulsivity. These individuals may be a subset of patients with lifestyles and drinking patterns less related to compensatory drinking behavior to manage symptoms of anxiety and mood disorders. Those with higher levels of anxiety and depression may require concurrent treatments to address alcohol use disorder‐related comorbidities.

The results obtained provide support for the likely responder statistical paradigm as a valuable tool for advancing precision medicine. This approach is able to identify a subgroup of patients likely to respond at least to a prespecified level in which the active treatment is demonstrably causally superior to placebo.

## Conflict of Interest

EL, CS, ZL, and CM receive research support from the National Institute on Alcohol Abuse (NIAAA) and Alcoholism, the National Institute of Mental Health (NIMH), and the Department of Defense; EL is a consultant to Catalyst Pharmaceuticals; MB has received support from the National Institute on Drug Abuse, the Heffter Research Institute, the Multidisciplinary Association of Psychedelic Studies, and Turnbull Family Foundation and has research support pending from B. More, Inc. and Mind Medicine, Inc. CM serves on the scientific advisory board and has equity in Receptor Life Sciences. He serves on the PTSD advisory board for Otsuka Pharmaceutical and has received support from the US Army Congressionally Directed Medical Res Program, the Steven and Alexandra Cohen Foundation, Cohen Veterans Bioscience, the Cohen Veterans Network, the Home Depot Foundation, the McCormick Foundation, the Robin Hood Foundation, and the City of New York.

## References

[acer14414-bib-0001] Anton RF , Myrick H , Wright TM , Latham PK , Baros AM , Waid LR , Randall PK (2011) Gabapentin combined with naltrexone for the treatment of alcohol dependence. Am J Psychiatry 168:709–717. 21454917 10.1176/appi.ajp.2011.10101436 PMC3204582

[acer14414-bib-0002] Breiman L (2001) Random forests. Mach Learn 45:5–32.

[acer14414-bib-0003] Buuren SV , Groothuis‐Oudshoorn K (2010) mice: Multivariate imputation by chained equations in R. J Stat Softw, 45(3), 1–67.

[acer14414-bib-0004] Chick J , Aschauer H , Hornik K (2004) Efficacy of fluvoxamine in preventing relapse in alcohol dependence: a one‐year, double‐blind, placebo‐controlled multicentre study with analysis by typology. Drug Alcohol Depend 74:61–70. 15072808 10.1016/j.drugalcdep.2003.11.012

[acer14414-bib-0020] European Medicines Agency (2019) Guideline on the investigation of subgroups in confirmatory clinical trials. https://www.ema.europa.eu/en/documents/scientific‐guideline/guideline‐investigation‐subgroups‐confirmatory‐clinical‐trials_en.pdf Published January 2019. Accessed August 15, 2019

[acer14414-bib-0006] Falk DE , Ryan ML , Fertig JB , Devine EG , Cruz R , Brown ES , Burns H , Salloum IM , Newport DJ , Mendelson J , Galloway G (2019) Gabapentin enacarbil extended‐release for alcohol use disorder: a randomized, double‐blind, placebo‐controlled, multisite trial assessing efficacy and safety. Alcohol Clin Exp Res 43:158–169. 30403402 10.1111/acer.13917 PMC6317996

[acer14414-bib-0007] Foster JC , Taylor JM , Ruberg SJ (2011) Subgroup identification from randomized clinical trial data. Stat Med 30:2867–2880. 21815180 10.1002/sim.4322 PMC3880775

[acer14414-bib-0008] Hansen BB (2008) The prognostic analogue of the propensity score. Biometrika 95:481–488.

[acer14414-bib-0009] Hartwell EE , Feinn R , Morris PE , Gelernter J , Krystal J , Arias AJ , Hoffman M , Petrakis I , Gueorguieva R , Schacht JP , Oslin D (2020) Systematic review and meta‐analysis of the moderating effect of rs1799971 in OPRM1, the mu‐opioid receptor gene, on response to naltrexone treatment of alcohol use disorder. Addiction. 10.1111/add.14975 PMC7340566 31961981

[acer14414-bib-0010] Hou J , Seneviratne C , Su X , Taylor J , Johnson B , Wang XQ , Zhang H , Kranzler HR , Kang J , Liu L (2015) Subgroup identification in personalized treatment of alcohol dependence. Alcohol Clin Exp Res 39:1253–1259. 26031187 10.1111/acer.12759 PMC4491003

[acer14414-bib-0011] Imbens GW , Rubin DB (2015) Causal inference in statistics, social, and biomedical sciences. ISBN: 978‐0‐52‐188588‐1. New York: Cambridge University Press.

[acer14414-bib-0012] Johnson BA , Ait‐Daoud N , Seneviratne C , Roache JD , Javors MA , Wang XQ , Liu L , Penberthy JK , DiClemente CC , Li MD (2011) Pharmacogenetic approach at the serotonin transporter gene as a method of reducing the severity of alcohol drinking. Am J Psychiatry 168:265–275. 21247998 10.1176/appi.ajp.2010.10050755 PMC3063997

[acer14414-bib-0013] Kranzler HR , Burleson JA , Brown J , Babor TF (1996) Fluoxetine treatment seems to reduce the beneficial effects of cognitive‐behavioral therapy in type B alcoholics. Alcohol Clin Exp Res 20:1534–1541. 8986200 10.1111/j.1530-0277.1996.tb01696.x

[acer14414-bib-0014] Kranzler HR , Feinn R , Morris P , Hartwell EE (2019) A meta‐analysis of the efficacy of gabapentin for treating alcohol use disorder. Addiction 114:1547–1555. 31077485 10.1111/add.14655 PMC6682454

[acer14414-bib-0027] Litten RZ , Falk D , Ryan M , Fertig J (2015) Heterogeneity of alcohol use disorder: understanding mechanisms to advance personalized treatment. Alcohol Clin Exp Res, 39:579–584. 25833016 10.1111/acer.12669

[acer14414-bib-0015] Mason BJ , Quello S , Goodell V , Shadan F , Kyle M , Begovic A (2014) Gabapentin treatment for alcohol dependence: a randomized clinical trial. JAMA Intern Med 174:70–77. 24190578 10.1001/jamainternmed.2013.11950 PMC3920987

[acer14414-bib-0028] Myrick H , Malcolm R , Randall PK , Boyle E , Anton RF , Becker HC , Randall CL (2009) A double‐blind trial of gabapentin versus lorazepam in the treatment of alcohol withdrawal. Alcohol Clin Exp Res 33:1582–1588. 19485969 10.1111/j.1530-0277.2009.00986.x PMC2769515

[acer14414-bib-0016] Neyman J (1923) Sur les applications de la theorie des probabilites aux experiences agricoles: essai des principes (Masters Thesis); Justification of applications of the calculus of probabilities to the solutions of certain questions in agricultural experimentation. Excerpts English translation (Reprinted). Stat Sci 5:463–472.

[acer14414-bib-0017] Pettinati HM , Volpicelli JR , Kranzler HR , Luck G , Rukstalis MR , Cnaan A (2000) Sertraline treatment for alcohol dependence: interactive effects of medication and alcoholic subtype. Alcohol Clin Exp Res 24:1041–1049. 10924008

[acer14414-bib-0018] Project MATCH Research Group (1997) Matching Alcoholism Treatments to Client Heterogeneity: Project MATCH Posttreatment drinking outcomes. J. stud. alcohol 58:7–2. 8979210

[acer14414-bib-0019] Project MATCH Research Group (1998) Matching alcoholism treatments to client heterogeneity: treatment main effects and matching effects on drinking during treatment. Project MATCH Research Group. J. Stud. Alcohol. 59:631–639. 9811084 10.15288/jsa.1998.59.631

[acer14414-bib-0021] Roberto M , Gilpin NW , O'Dell LE , Cruz MT , Morse AC , Siggins GR , Koob GF (2008) Cellular and behavioral interactions of gabapentin with alcohol dependence. J Neurosci 28:5762–5771. 18509038 10.1523/JNEUROSCI.0575-08.2008 PMC2493536

[acer14414-bib-0022] Rosenbaum PR , Rubin DB (1983) The central role of the propensity score in observational studies for causal effects. Biometrika 70:41–55.

[acer14414-bib-0023] Rubin DB (2005) Causal inference using potential outcomes: Design, modeling, decisions. Journal of the American Statistical Association 100:322–331.

[acer14414-bib-0024] Rubin DB (1974) Estimating causal effects of treatments in randomized and nonrandomized studies. J Educ Psychol 66:688.

[acer14414-bib-0025] Sills GJ , Rogawski MA (2020) Mechanisms of action of currently used antiseizure drugs. Neuropharmacology:107966. 32120063 10.1016/j.neuropharm.2020.107966

[acer14414-bib-0026] Wallach JD , Krystal JH , Ross JS , O’Malley SS . 2020 Characteristics of Ongoing Clinical Trials for Alcohol Use Disorder Registered on ClinicalTrials.gov. JAMA Psychiatry. Published online May 27, 2020. 10.1001/jamapsychiatry.2020.1167 PMC7254444 32459295

